# Supplementary material for: Interspecies Transmission from Pigs to Ferrets of Antigenically Distinct Swine H1 Influenza A Viruses with Reduced Reactivity to Candidate Vaccine Virus Antisera as Measures of Relative Zoonotic Risk
Source: Viruses. 2022 Oct 29;14(11):2398. doi: 10.3390/v14112398 (PMC9698830; doi:10.3390/v14112398)
Supplement: Supplementary file 1 [file viruses-14-02398-s001.zip › viruses-1969528-supplementary.pdf]

Interspecies Transmission from Pigs to Ferrets of Antigenically Distinct Swine H1 Influenza A Viruses with Reduced Reactivity to Candidate Vaccine Virus Antisera as Measures of Relative Zoonotic Risk

J. Brian Kimble[1#], Carine K. Souza[1], Tavis K. Anderson[1], Zebulun W. Arendsee[1], David E. Hufnagel[1], Katharine M. Young[1], Nicola S. Lewis[2], C. Todd Davis[3], Sharmi Thor [3], Amy L. Vincent Baker[1,*]

[1]Virus Prion Research Unit, National Animal Disease Center, USDA-ARS, Ames IA.

[2]Department of Pathology and Population Sciences, Royal Veterinary College, University of London, Hertfordshire, UK.

[3]Influenza Division, National Center for Immunization and Respiratory Diseases, Centers for Disease Control and Prevention, Atlanta GA.

*Address correspondence to Amy Vincent Baker, amy.vincent@usda.gov

[#]Current address: Foreign Arthropod-borne Animal Disease Research Unit, National Bio and Agro-Defense Facility, USDA-ARS, Manhattan, KS

SUPPLEMENTAL MATERIAL

Table S1. Amino acid differences between 1A.1.1.3 clade consensus, the within-clade CVV A/Ohio/24/2017, and the clade representative viruses A/swine/North Caroline/A02245416/2020 and A/swine/Texas/A02245420/2020. The clade includes a two amino acid deletion and H1 numbering and corresponding 1A.1.1.3 sequential numbering are presented.

1A.1.1.3	H1 numbering	1A.1.1.3 numbering	1A.1.1.3 consensus	A/Ohio/24/2017 CVV	A/swine/NC/A02245416/2020	A/swine/Tx/A02245420/2020
	14	14	D			E
	48	48	A			P
	68	68	E		G	
	119	119	M	I		I
	126	126	Y	H		
	132	130	V			E
	138	136	D			Y
	141	139	A			R
	142	140	S			G
	149	147	M	I		
	156	154	D	N		
	168	166	N			D
	170	168	R			G
	176	174	L		I	
	209	207	E			K
	216	214	T			I
	224	222	T			A
	232	230	T			A
	235	233	E		K	
	252	250	K			R
	264	262	G		S	
	310	308	T			R
	311	309	K			R
Differences from consensus			-	4	4	15
Differences from CVV			4	-	8	16

Table S2. Amino acid differences between 1A.3.3.2 clade consensus, the within-clade human seasonal vaccine A/Idaho/07/2018, and the clade representative A/swine/Utah/A02432386/2019.

1A.3.3.2	H1 numbering	1A.3.3.2 consensus	A/Idaho/07/2018	A/swine/UT/A02432386/2019
	47	V		I
	74	R		S
	164	T		S
	183	P		S
	205	R		K
	216	T		S
	260	D	N	N
	295	I		I
Difference from consensus		-	1	8
Difference from vaccine		1	-	7

Table S3. Amino acid differences between 1A.3.3.3 clade consensus, the within-clade CVV A/Ohio/09/2015, and the clade representative virus A/swine/Minnesota/A02245409/2020.

1A.3.3.3	H1 numbering	1A.3.3.3 consensus	A/Ohio/09/2015 CVV	A/swine/MN/A02245409/2020
	2	T	K	
	3	L	I	
	36	R	K	
	71	S	A	
	83	S		P
	84	N	S	
	86	E	N	
	113	R	K	
	120	T		A
	127	D	E	
	137	P		S
	146	R	K	
	149	V	I	
	153	Q	K	
	155	G	E	
	161	V	I	
	163	K	I	
	166	I	T	
	169	K	R	R
	170	E	G	
	183	S	P	
	186	A	T	
	192	K	Q	
	196	D	N	
	197	A	S	
	205	K	R	
	221	D	G	
	250	A	V	
	269	D	E	
	271	S	P	
Differences from consensus		-	27	4
Differences from CVV		27	-	29

Table S4. Amino acid differences between 1B.2.1 clade consensus, the within-clade CVVs A/Ohio/35/2017 and A/Michigan/383/2018, and the clade representative virus A/swine/Illinois/A02139356/2018.

1B.2.1	H1 numbering	1B.2.1 consensus	A/Ohio/35/2017 CVV	A/Michigan/383/2018 CVV	A/swine/IL/A02139356/2018
	36	S	T		
	71	T	I	N	
	72	S	P		
	83	S	P		
	96	E	T		
	113	K	R		
	120	K	D		
	121	S	G		
	129	T	S		
	156	N	G		
	169	E	K		K
	170	E		G	
	173	I	V	V	
	185	M	I		
	189	R	K		
	208	R	K		
	209	R	K		
	216	K	R		
	259	K			R
	260	G		S	
	261	F	S		
	277	T	A		
	289	S	D		
	310	A	T		
Differences from consensus		-	21	4	2
Difference from OH/35 CVV		21	-	22	21
Difference from MI/383 CVV		4	22	-	6

Table S5. Amino acid differences between 1B.2.2.1 clade consensus, the within-clade CVV A/Iowa/32/2016 and the clade representative virus A/swine/Iowa/A02478968/2020.

1B.2.2.1	H1 numbering	1B.2.2.1 consensus	A/Iowa/32/2016 CVV	A/swine/IA/A02478968/2020
	19	V	L	
	74	K		E
	84	N		D
	96	A	T	
	132	V	K	
	141	K	E	
	166	K		E
	168	D	E	
	237	G		R
	273	D		N
	277	A		T
Differences from consensus		-	5	6
Differences from CVV		5	-	11

Table S6. Amino acid differences between 1B.2.2.2 clade consensus and the clade representative virus A/swine/Colorado/A02245414/2020.

1B.2.2.2	H1 numbering	1B.2.2.2 consensus	A/swine/CO/A02245414/2020
	36	S	N
	50	L	I
	72	S	P
	85	S	P
	176	L	I
	208	G	E
	228	N	K
	236	P	A
	249	I	V
	271	P	S
	274	E	K
Differences from consensus		-	11

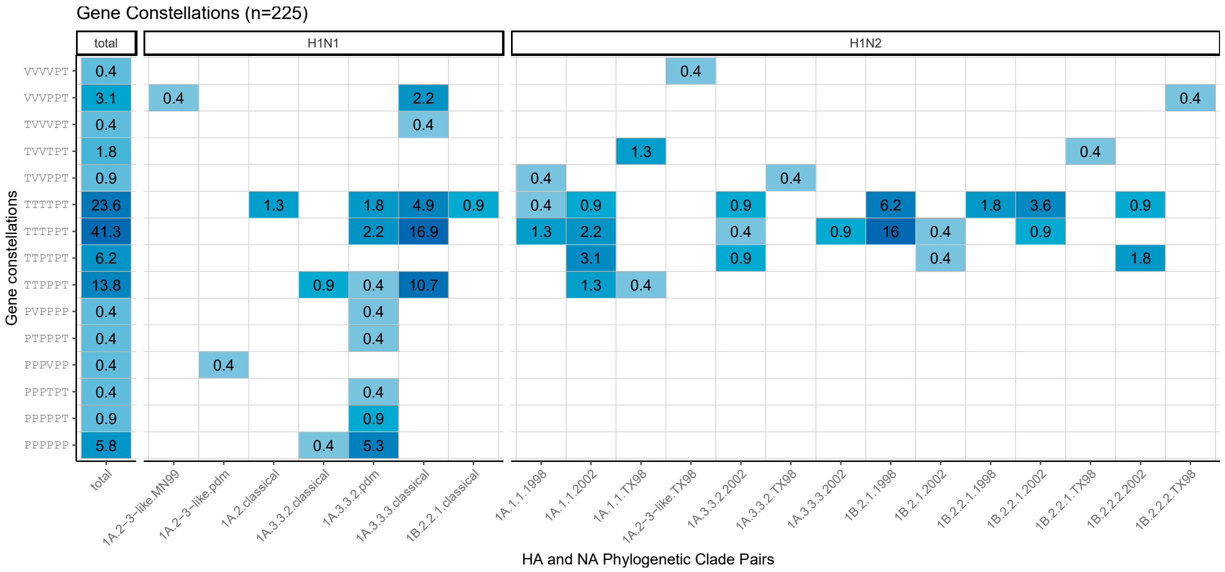

Figure S1. Proportions of detected H1N1 and H1N2 influenza A virus in swine collected in 2020 in the USDA influenza A virus in swine surveillance system. The x-axis reflects the paired genetic clade of hemagglutinin (HA) and neuraminidase (NA), and the y-axis reflects the evolutionary lineage (triple-reassortant, T; H1N1pdm09, P; live attenuated vaccine virus associated, V) of the internal genes in the order of PB2-PB1-PA-NP-M-NS.
